# Managing Conflict Case: The Difficult Consultant

**DOI:** 10.21980/J8.53815

**Published:** 2025-12-31

**Authors:** Amrita Vempati, Suzanne Bentley, Anita Rohra, Daniela Ortiz, David Fernandez, Shagun Berry, Colleen Donovan, Nicole Novotny, Stephanie Cohen, Stephanie Stapleton, Tiffany Moadel

**Affiliations:** 1 Creighton School of Medicine Phoenix, Department of Emergency Medicine, Phoenix, AZ; 2 NYC Health + Hospitals, Department of Emergency Medicine, Brooklyn, NY; 3 Baylor College of Medicine, Department of Emergency Medicine, Houston, TX; 4 Mount Sinai Hospital, Department of Emergency Medicine, Brooklyn, NY; 5 Rush University Medical Center, Department of Emergency Medicine, Chicago, IL; 6 Rutgers Robert Wood Johnson Medical School, Department of Emergency Medicine, New Brunswick, NJ; 7 Ochnser Health System, Department of Emergency Medicine, New Orleans, LA; 8 University of Central Florida, Department of Emergency Medicine, Orlando, FL; 9 Boston University/Boston Medical Center, Department of Emergency Medicine, Boston, MA; 10 Zucker School of Medicine at Hofstra/Northwell, Department of Emergency Medicine, Hempstead, NY

## Abstract

**Audience:**

This communication case is intended for emergency medicine (EM) resident physicians and junior faculty preparing for the ABEM Certifying Exam.

**Introduction:**

Conflict management is a common and significant challenge in the emergency department (ED), with potential negative impact on both physicians and patients. At the individual level, hospital-based conflicts can lead to emotional stress, impaired concentration, decreased trust among providers, and feelings of dehumanization.^1^ For patients, conflicts between physicians can compromise the quality of care and contribute to delays in treatment.^1^ In the ED, conflict often arises between emergency physicians (EPs) and various stakeholders, including patients, family members, nursing staff, hospitalists, and consultants.^2^ Recognizing its importance, the American Board of Emergency Medicine (ABEM) has identified conflict management as a core competency for emergency medicine residents and has incorporated it into the oral certification examination.^3^ This oral examination case was developed to help train EM residents in managing conflict—specifically between an emergency physician and a consultant—in a structured environment.

**Educational Objectives:**

By the end of the session, the learner should be able to: 1) review format and have become familiar with an ABEM Certifying Exam conflict management communication case, 2) demonstrate the ability to initiate the consultation call, establish rapport, and present a concise, evidence-based summary of the patient’s STEMI findings, 3) recognize concerns raised by the cardiologist and respond with an empathetic acknowledgment (eg, validating workload, uncertainty, or resource constraints) to support a collaborative tone, 4) articulate differing viewpoint by referencing objective clinical data (eg, ST-segment elevations, ongoing chest pain, risk from delays) when conflict is encountered and justify why urgent catheterization lab activation is indicated, and 5) identify shared goals in optimizing patient care (reducing myocardial damage and preventing deterioration) and use these to negotiate a mutually acceptable plan.

**Educational Methods:**

This is a standardized patient scenario in alignment with the expected Managing Conflict case format of the ABEM Certifying Exam. The benefits of this educational modality are that it allows for direct observation and assessment of a learner’s ability to communicate with a consultant and to observe the use of conflict resolution strategies.

**Research Methods:**

We used an iterative case trialing process with a convenience sample of EM residents. Feedback from each site was used to make iterative changes to the case material. Facilitators completed a Simulation Scenario Evaluation Tool^4^ (SSET) survey during the written and alpha trials to evaluate the quality of key simulation elements. Facilitators and learners used modified usability surveys to evaluate cases during the beta trials. All surveys were completed anonymously using 5-point Likert scales and free text responses. All data were collected using Qualtrics (https://www.qualtrics.com) and analyzed using Excel (Microsoft, Redmond, WA). The Boston University Institutional Review Board reviewed the project and deemed it exempt.

**Results:**

A total of 14 senior resident learners and 4 facilitators evaluated the case. The SSET data were largely positive; case objectives, key actions and materials were clear. The facilitators found the case was easy to use, and thought others would feel similarly. They felt confident using the case and would like to use this case for ABEM certifying exam practice. Over 90% of residents found both the written and verbal case materials to be clear. Ninety percent of residents reported that the experience was helpful practice for the ABEM certifying exam.

**Discussion:**

The case was well-received by both learners and facilitators due to its realism and similarity to real-life encounters and appears to be a useful preparatory tool for the ABEM Certifying Exam’s Managing Conflict case because it allows learners to practice conflict resolution skills.

**Topics:**

Conflict resolution, communication, ABEM Certifying Exam.

## USER GUIDE

**Table:** 

List of Resources:	
Abstract	43
User Guide	45
For Examiner Only	47
Certifying Exam Assessment	52
Stimulus	54
Debriefing and Evaluation Pearls	56

**Learner Audience:**

This case is appropriate for interns, junior and senior residents, and junior EM Attendings preparing for ABEM Certifying Exams.

**Time Required for Implementation:**

Case: 10 minutes

Debriefing: 10 minutes

**Recommended number of learners per instructor:**

Ideally one learner per instructor; however, another learner may be able to learn by observing the simulation and participating in the debriefing.

**Topics:**

Conflict management, communication, ABEM Certifying Exam

**Objectives:**

By the end of the session, the learner should be able to:

1. Review format and become familiar with an ABEM Certifying Exam conflict management communication case.
2. Demonstrate the ability to initiate the consultation call, establish rapport, and present a concise, evidence-based summary of the patient’s STEMI findings.
3. Recognize concerns raised by the cardiologist and respond with an empathetic acknowledgment (eg, validating workload, uncertainty, or resource constraints) to support a collaborative tone.
4. Articulate differing viewpoint by referencing objective clinical data (eg, ST-segment elevations, ongoing chest pain, risk from delays) when conflict is encountered and justify why urgent catheterization lab activation is indicated.

### Linked objectives, methods and results

These objectives were selected to align with the ABEM Certifying Exam Managing Conflict case type. In this standardized patient case, the participant receives clinical information about a patient presenting with substernal chest pain, diaphoresis, and an EKG demonstrating clear ST-segment elevations in the anterior leads. The participant is instructed to speak with the on-call cardiologist to request emergent cardiac catheterization. At the start of the interaction, the learner is expected to open the consultation professionally, establish rapport, and deliver a concise, evidence-based presentation of the patient’s condition (Objective 2). During the scenario, the cardiologist responds with skepticism, questioning the participant’s ability to interpret the EKG, minimizing the patient’s symptoms, and suggesting alternative explanations for the patient’s condition. During this phase, the participant must recognize the consultant’s concerns, acknowledge them empathetically, and validate their viewpoint to maintain a collaborative tone (Objective 3). As the consultation progresses, the cardiologist challenges the need for emergent catheterization, asserting that the EKG changes are nonspecific or that the patient could be managed medically. At this point, the participant must clearly and respectfully articulate their differing clinical opinion by citing objective findings such as ongoing chest pain, dynamic ST-segment elevation, hemodynamic risk, and the dangers of delayed re-perfusion (Objective 4). To resolve the conflict, the participant must then identify shared goals such as preventing myocardial injury and ensuring patient safety and use those mutual priorities to negotiate a unified management approach (Objective 5). The scenario concludes when the participant successfully guides the cardiologist towards agreement on activating the catheterization lab or reaches a mutually accepted plan towards STEMI best practices.

### Recommended pre-reading for instructor

- Westafter L. *Why Can’t We Be Friends – Conflict in Emergency Medicine. SGEM#445.* Podcast: The Skeptics Guide to Emergency Medicine. Published July 6, 2024. Accessed December 3, 2025. https://thesgem.com/2024/07/sgem445-why-cant-we-be-friends-conflict-in-emergency-medicine/
- Garmel GM. Conflict resolution in emergency medicine. In: Adams, JG, editor. *Emergency Medicine: Clinical Essentials*. (2nd Ed. Philadelphia: Saunders/Elsevier, 2013:2171–2185.
- Back AL, Arnold RM. Dealing with conflict in caring for the seriously ill: “it was just out of the question.” *JAMA*. 2005;293(11):1374–81. doi:10.1001/jama.293.11.1374. PMID: 15769971

### Results and tips for successful implementation

The case was tested on a total of 14 senior resident learners and four EM faculty facilitators using an iterative case trialing process at multiple sites with a convenience sample of EM residents. Learners and facilitators provided experience feedback on the quality of case elements via anonymous surveys using Likert scale items and free form comments. We used a modified SSET to evaluate the written and alpha round of cases. We used modified usability surveys for the two beta trialing rounds. Likert scale items were evaluated on a range of 1 to 5, where 1 = strongly disagree, 2 = disagree, 3 = neutral, 4 = agree, and 5 = strongly agree. All data were collected using Qualtrics (https://www.qualtrics.com) and analyzed using Excel (Microsoft, Redmond, WA). The Boston University Medical Center Institutional Review Board reviewed the project and deemed it exempt.

Data from the surveys demonstrated favorable marks among both learners and facilitators regarding the quality and usability of the case materials. Among the four senior resident learners, three indicated agreement or strong agreement that the written materials were clear and the case was helpful practice for the ABEM Certifying Exam. Regarding clarity, one learner felt that the case should specify that there is no other nearby ST-elevation MI (STEMI) center that the patient can be transported to. The Task Statement section of the Candidate Task Sheet was updated to reflect this pertinent point in response to this feedback. One EM faculty facilitator strongly agreed that the case was easy to use, its materials were well integrated, and thought others would feel similar. They also felt confident using the case and would like to use this case for ABEM Certifying Exam practice.

The alpha round of case trialing was performed at an academic EM residency program, where one facilitator (the same facilitator as in round one) and one resident completed surveys. During the first round of beta trialing the case, a simulation-trained EM faculty member at the 2025 Society for Academic Emergency Medicine (SAEM) Annual Meeting in Philadelphia, PA, tested the case with three EM senior resident learners. A second round of beta trialing was performed at an additional academic EM residency program, where three facilitators and 11 residents completed modified usability surveys. Beta trialing evaluation scores were generally positive; SAEM mean of 4.5 and second beta site mean 4.9.

This case is best implemented in a private room devoid of distractions with one participant, one standardized patient, and the facilitator present.

## Figures and Tables

**Table:** Managing Conflict Candidate Task Sheet

**CASE PARAMETERS**	
This is a 10-minute case. You will interact with a cardiologist. Pertinent physical exam and diagnostic findings are provided. Physical exam findings are normal, unless otherwise stated. Laboratory tests have been sent, but have not yet resulted. You gave the patient 324 mg of aspirin and told him that he will need to be taken to the cardiac catheterization lab for an ST-elevation MI (STEMI). Your hospital has a cardiac catheterization lab. The next closest STEMI center is over 100 miles away.	
**PATIENT INFORMATION**	
**Patient name**	Joshua Burns
**Age**	35
**Gender**	Male
**Presenting Complaint**	Chest pain
**Past Medical History**	None
**Medication**	None
**Allergies** No	known drug allergies
**Vital Signs on ED Arrival**	BP 98/52, P 110, RR 20, T 37.3° C, O2 Sat 98%
**PERTINENT FINDINGS**	
The patient is a 35-year-old male who presents with “crushing” anterior chest pain. The pain began one hour ago while lifting a heavy piece of equipment. He reports it as 10/10, radiating down his left arm. He reports the pain has progressively worsened over the past two weeks. He has had episodes of chest pain that would improve with rest; however, this episode has not yet resolved. The patient appears diaphoretic and uncomfortable. Laboratory tests have been sent; however, they are not yet available. Serial ECGs obtained in the ED demonstrate evolving ST-segment elevations in the anterolateral leads, without reciprocal changes. A bedside echocardiogram reveals wall motion abnormalities involving the septum and lateral wall of the left ventricle. A chest x-ray appears normal. While being monitored in the ED, the patient was observed on telemetry to have multiple runs of non-sustained ventricular tachycardia. The patient is an active one pack-per-week cigarette smoker. The patient’s father died of an MI at the age of 40.	
**TASK STATEMENT**	
Your task is to speak with the cardiologist and ask them to take the patient for a cardiac catheterization.	

## CERTIFYING EXAM ASSESSMENT

*Managing Conflict Case: The Difficult Consultant*

Learner: _________________________________________

**Facilitator Guidelines**

- Up-score if the learner keeps an even voice, calm body language, and objective language.
- Down-score if the learner attempts to change their original plan.
- while seeking help from their superiors may be the ultimate plan for the learner, they should not attempt to use notification of the hospital/department administration to threaten the consultant. Down-score if this happens.

**Introduction & Communication of Plan**

□ Establishes rapport with the consultant
□ Clearly and succinctly presents the clinical situation
□ Communicates rationale

**Empathy & Validation**

□ Uses empathetic language/tone
□ Acknowledges the consultant’s concerns directly
□ Demonstrates active listening (eg, reflective statements, pauses)

**Recognition of Differing Viewpoints**

□ Identifies the consultant’s alternative perspective
□ Verbalizes understanding of differing opinions
□ Remains respectful and non-confrontational

**Shared Goals & Collaborative Mindset**

□ Redirects conversation toward shared goals
□ Frames discussion around what is best for the patient
□ Attempts to find common ground or compromise

**Resolution & Agreement**

□ Summarizes the agreed-upon plan
□ Confirms mutual understanding
□ Maintains professionalism throughout the encounter

**Summative and formative comments:**

### Stimulus Inventory

**Candidate Task Sheet**
